# Estimating individualized gonadotropin starting doses in women with PCOS: a target-driven machine learning model for GnRH-antagonist stimulation

**DOI:** 10.1186/s12884-026-09783-x

**Published:** 2026-08-10

**Authors:** Wenjia Meng, Ye Pan, Moxuan Zhang, Ziyi Song, Zerui Xiao, Rong Liang, Xiao Shi, Hongchu Bao, Lili Zhuang, Qun Lu

**Affiliations:** 1 Medical Center for Human Reproduction, Beijing Chao-Yang Hospital, Capital Medical University, Beijing, 100020 China; 2 Beijing Neurosurgical Institute, Beijing Tiantan Hospital, Capital Medical University, Beijing, 100070 China; 3 Center of Reproductive Medicine, Peking University People’s Hospital, Beijing, 100044 China; 4 Reproductive Medicine Centre, Yuhuangding Hospital of Yantai, Affiliated Hospital of Qingdao University, Yantai, 264000 China

**Keywords:** PCOS, Machine learning, Gn starting dose, BMI, Cumulative live birth rate

## Abstract

**Background:**

Women with polycystic ovary syndrome (PCOS) are prone to excessive ovarian response and ovarian hyperstimulation syndrome (OHSS) during controlled ovarian stimulation, whereas overly conservative gonadotropin (Gn) dosing reduces oocyte yield and efficiency. This study aimed to develop a target-driven machine learning model for estimating individualized Gn starting doses in women with PCOS.

**Methods:**

This retrospective, two-center study analyzed 976 PCOS women undergoing in vitro fertilization-embryo transfer using GnRH-antagonist protocols. Patients were categorized by oocyte yield (< 7, 7–16, > 16). Ovarian sensitivity (OS), defined as retrieved oocytes per unit of Gn starting dose, was modeled as a continuous response metric linking baseline characteristics with Gn requirements. Data were partitioned into training, validation, and test sets (60:20:20). Parallel random forest (RF) and least absolute shrinkage and selection operator (LASSO) regression identified core predictors within the training set. Model accuracy was evaluated via R^2^, RMSE, and MAE.

**Results:**

The 7–16 oocyte-yield category showed a more favorable balance of reproductive outcomes, OHSS risk, time to live birth, and costs. Body mass index (BMI), anti-Müllerian hormone (AMH), basal follicle-stimulating hormone (bFSH), and antral follicle count (AFC) were retained as final predictors. The RF model robustly predicted continuous OS in the independent test set, achieving an R^2^ of 0.860 (95% CI: 0.809, 0.901), an RMSE of 0.254 (95% CI: 0.218, 0.296), and an MAE of 0.141 (95% CI: 0.127, 0.156). Post-hoc dose-concordance analysis in the 7–16 category showed model-derived doses correlated with historical prescriptions. Simulated dose-adjustment analyses generated upward recommendations for < 7 oocytes and downward recommendations for > 16 oocytes. An online “Gn Calculator” was developed for clinical decision support.

**Conclusions:**

By predicting observed OS via BMI, AMH, bFSH, and AFC, this target-driven RF framework calculates individualized Gn starting doses for women with PCOS. The Gn Calculator facilitates clinical application and may help guide patients toward optimal outcomes by targeting an ideal oocyte yield.

**Supplementary Information:**

The online version contains supplementary material available at https://doi.org/10.1186/s12884-026-09783-x.

## Background

Polycystic ovary syndrome (PCOS) is a complex endocrine, metabolic, and psychological disorder that affects approximately 5–20% of women in their reproductive years worldwide. This syndrome is characterized by a wide range of symptoms, including clinical or biochemical indicators of hyperandrogenism, ovulatory dysfunction, and polycystic ovarian morphology, often accompanied by insulin resistance and obesity [1–3]. PCOS is also one of the leading causes of anovulatory infertility, and women with PCOS often pursue in vitro fertilization-embryo transfer (IVF-ET) treatments to improve their chances of conception after unsuccessful attempts with ovulation induction and/or intrauterine insemination (IUI) therapies [4, 5]. During controlled ovarian stimulation (COS), women with PCOS are prone to exaggerated ovarian responses because of high ovarian reserve and increased sensitivity to exogenous gonadotropin (Gn), which increases the risk of high response, ovarian hyperstimulation syndrome (OHSS), fresh embryo transfer cancellation, and unplanned freeze-all strategies. Conversely, overly conservative Gn starting doses may lead to insufficient follicular recruitment, reduced oocyte yield, fewer available embryos, and lower treatment efficiency. Therefore, determining an appropriate Gn starting dose is a critical clinical step for balancing efficacy and safety in PCOS patients undergoing gonadotropin-releasing hormone (GnRH) antagonist stimulation.

Balancing these therapeutic extremes necessitates a targeted clinical approach. Previous studies in PCOS and assisted reproductive technology populations have suggested that retrieval of approximately 7–16 oocytes may represent a clinically desirable range associated with favorable reproductive outcomes [6–9]. Importantly, the < 7, 7–16, and > 16 oocyte categories applied in this study serve strictly as predefined target-yield brackets to guide dose estimation; they are not intended to challenge or redefine the established diagnostic criteria for poor, normal, or high ovarian response, such as the Bologna or POSEIDON classifications [10, 11].

Although GnRH-antagonist protocols and freeze-all strategies are effective approaches for reducing OHSS risk in high responders, they do not eliminate the need for accurate gonadotropin dose selection at stimulation initiation. Freeze-all strategies may prolong treatment, increase healthcare costs, and have been associated in some studies with specific obstetric outcomes after frozen-thawed embryo transfer (FET), including hypertensive disorders of pregnancy and large-for-gestational-age offspring [12–14]. Therefore, when clinically safe and appropriate, avoiding excessive ovarian response may reduce the need for unplanned freeze-all strategies and preserve the possibility of fresh embryo transfer, while also reducing treatment burden.

Determining the optimal starting dose remains challenging. Recent large-scale studies have classified PCOS into distinct data-driven subtypes, highlighting the profound heterogeneity of the syndrome and underscoring the critical importance of achieving an ideal ovarian response to mitigate subtype-specific risks [15]. Because suboptimal ovarian responses are associated with compromised reproductive outcomes, accurately determining the Gn starting dose is essential. While existing models use conventional markers, such as age, anti-Müllerian hormone (AMH), antral follicle count (AFC), and basal follicle-stimulating hormone (bFSH), their applicability to PCOS-specific, target-driven dose estimation remains limited [16–20]. Many rely on linear frameworks, incorporate excessive variables that impede bedside application, or omit body mass index (BMI), a crucial metabolic determinant of Gn pharmacokinetics in PCOS [21, 22]. Therefore, integrating these baseline indicators to construct a novel starting dose model through a non-linear algorithm is the key to achieving individualized dosing.

Ovarian sensitivity (OS), defined as the ratio of retrieved oocytes to the Gn starting dose, serves as the quantitative bridge linking baseline patient characteristics to the exogenous Gn requirements for achieving an optimal oocyte yield [18, 19, 23]. By modeling OS as a continuous observed response metric rather than predicting administered doses directly, this approach significantly reduces the risk of simply reproducing historical prescribing habits. In the present study, we integrated these baseline indicators to construct a non-linear machine learning model anchored by OS. Utilizing this core parameter, our target-driven algorithm calculates individualized Gn starting doses, thereby maximizing reproductive success while minimizing stimulation risks for women with PCOS.

## Methods

### Participants and study design

This bi-center retrospective study included 976 women with PCOS who underwent IVF/intracytoplasmic sperm injection (ICSI) cycles utilizing GnRH-antagonist protocols. Participants were recruited from the Center of Reproductive Medicine, Peking University People’s Hospital (*n* = 237, January 2016-December 2019) and the Reproductive Medicine Center at Yuhuangding Hospital of Yantai (*n* = 739, January 2015-April 2020). The study protocol was approved by the Institutional Review Board of Peking University People’s Hospital (No. 2020PHB142-02).

The diagnosis of PCOS was based on the Rotterdam criteria [2], which require the presence of at least two out of three key indicators, including oligo- or anovulation, clinical and/or biochemical signs of hyperandrogenemia, and polycystic ovaries. Polycystic ovarian morphology was defined by the presence of 12 or more follicles in each ovary measuring 2–9 mm in diameter and/or an increased ovarian volume (10 ml or more). Cases with missing data or other causes of hyperandrogenemia and ovulatory dysfunction, such as congenital or atypical adrenal hyperplasia, thyroid disease, Cushing’s syndrome, and androgen-secreting tumors, were excluded. Patients with a history of unilateral oophorectomy, recurrent miscarriages, congenital or acquired uterine malformations, and abnormal karyotype results in either or both partners were also excluded. The collection of patient baseline data was documented in Section S1 of the supplementary appendix (SA).

### COS protocols and oocyte collection

All patients were administered GnRH-antagonist regimens, with exogenous Gn administration beginning on days 2–3 of menstruation. Fertility specialists implemented individualized COS protocols, considering the ovarian reserve of each patient and other factors. The PCOS population was advised to commence treatment with a dosage of 100–150 IU [24]. The exogenous Gn utilized included recombinant human FSH for injection (Gonal-F, Follitropin alfa from Merck Serono, Darmstadt, Germany, and Puregon, Follitropin beta from MSD, Kenilworth, NJ, USA) and human menopausal gonadotropin (HMG) for injection (Lizhu Pharmaceutical Factory, Zhuhai, China). When the growing follicles attained a diameter of 12–14 mm and exhibited a serum estradiol (E_2_) level exceeding 200–300 pg/ml, the GnRH-antagonist treatment was initiated. The trigger time was determined when at least two follicles reached 18 mm, or the average diameter of three follicles reached 17 mm, while also considering progesterone and estradiol levels. Human chorionic gonadotropin (hCG), or a GnRH-agonist, or GnRH-agonist combined with 2000 IU hCG, was used to trigger follicle maturation. Transvaginal oocyte retrieval took place 34–36 h after the trigger.

### IVF-ET and pregnancy outcomes

The fertilization process, dependent on the quality and quantity of sperm and the capacity for oocyte fertilization, can be conducted via conventional insemination or ICSI. Embryos at the cleavage stage or blastocysts undergo evaluation on day 3 or 5–6 post-oocyte retrieval. One or two top-scoring embryos were selected for fresh transfer, while surplus embryos were subjected to cryopreservation or cultured further to the blastocyst stage. All embryo freezing may be considered in cases of potential OHSS risk (the details in Section S2 in SA), an unfavorable endometrial environment, premature elevation of progesterone (serum progesterone level above 1.5 ng/ml on the trigger day), or other conditions. Subsequent FET, utilizing an appropriate protocol such as natural cycle FET or hormone replacement therapy FET, can be scheduled at a suitable time.

Biochemical pregnancy was defined as a serum hCG concentration > 10 mIU/mL 14 days after embryo transfer. Clinical pregnancy was defined as ultrasonographic visualization of at least one intrauterine gestational sac 35 days after embryo transfer. Progesterone was administered after embryo transfer for luteal-phase support and was tapered between 10 and 12 weeks of gestation. Live birth was defined as delivery of at least one live-born infant at or beyond 28 weeks of gestation. The cumulative pregnancy rate (CPR) was defined as the proportion of oocyte retrieval cycles resulting in at least one clinical pregnancy across all associated embryo transfer cycles, including fresh and/or subsequent frozen-thawed embryo transfers using embryos derived from that retrieval. The cumulative live birth rate (CLBR) was defined as the proportion of oocyte retrieval cycles resulting in at least one delivery with at least one live-born infant across those embryo transfer cycles. For each cumulative outcome, follow-up continued until the first occurrence of the relevant outcome, exhaustion of all embryos derived from the retrieval, or the end of follow-up, whichever occurred first. The denominator for both CPR and CLBR was the total number of eligible oocyte retrieval cycles, with all fresh and subsequent frozen-thawed embryo transfer cycles using embryos derived from each retrieval included in the cumulative assessment.

### Machine learning modeling strategy and dose derivation

As a stable and sensitive indicator, OS was selected as the continuous modeling target to quantitatively bridge baseline characteristics with Gn requirements [25]. Prior to modeling, raw OS values underwent log-transformation to correct for the right-skewed distribution.

The entire dataset (*n* = 976) was randomly partitioned (60:20:20) for training, validation, and testing as pre-defined. To rigorously prevent data leakage, all feature selection procedures and subsequent model training were executed exclusively within the training set. Candidate predictors-including baseline clinical, embryological, and endocrine parameters, among others-were screened using a robust dual-screening approach for predicting OS, which combined RF and least absolute shrinkage and selection operator (LASSO) algorithms to construct a parsimonious model. The RF algorithm assessed feature importance based on the percentage Increase in Mean Squared Error (%IncMSE), and the number of trees (ntree) was set to 500. Concurrently, LASSO regression was applied to penalize absolute coefficients. To prevent overfitting and build a simple, parsimonious model, the optimal penalty parameter ($\:\lambda$) was determined using the 1-standard error (1-SE) rule via 10-fold cross-validation. Only consensus variables robustly highlighted by both algorithms were retained for final modeling.

Subsequently, a series of RF regression models (incorporating 16, 8, and 4 variables, respectively) were constructed to predict continuous OS. The predictive algorithms were implemented using the randomForest and caret packages in R software. To optimize the model architecture, hyperparameter tuning was executed via a grid search strategy combined with 10-fold cross-validation within the training set. For the final streamlined 4-variable model, the number of decision trees (ntree) was maintained at 500, and the optimal number of variables sampled at each split (mtry) was determined to be 3. Model performance was evaluated across all datasets using the coefficient of determination (R^2^), root mean square error (RMSE), and mean absolute error (MAE). 

To bridge biological prediction with clinical intervention, a mathematical dose derivation was applied [18, 19, 23]. Since the continuous modeling target was log-transformed, the model output represents $\:\mathrm{ln}\left(OS\right)$. We derived the Gn starting dose using the ratio formula:

$$Gn\;starting\;dose=\frac{Target\;oocytes}{Predicted\;OS}$$

**Fig. 1:**
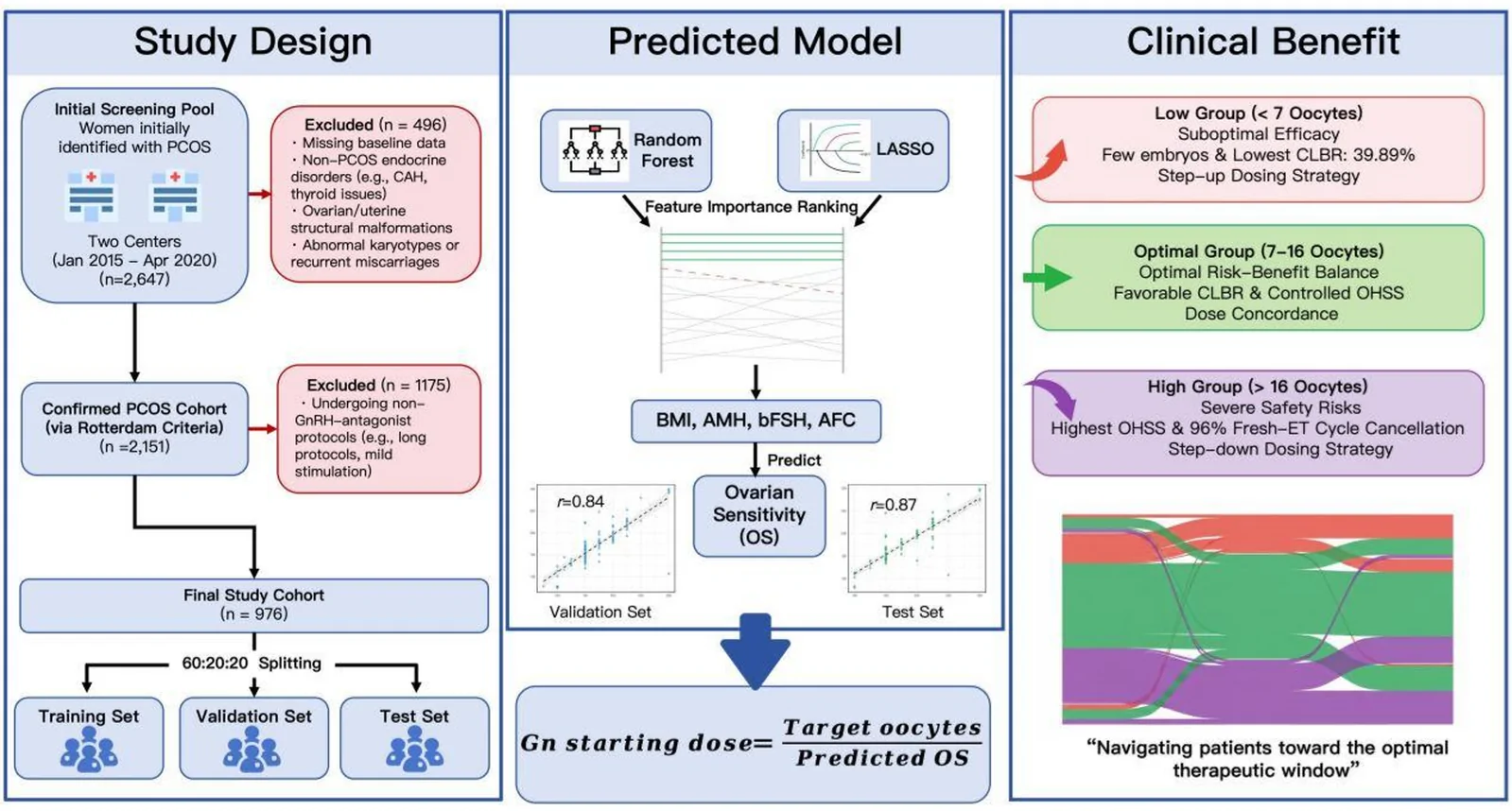
Overall study design and clinical framework

By inputting a physician-defined target oocyte yield (numerator) and utilizing the model-predicted OS (denominator), the model functions as a dynamic navigator. To validate the clinical reliability of the model, we assessed dose concordance within the optimal group (7–16 oocytes) [6] across both validation and test sets. In this group, we utilized the actual retrieved oocyte count as the target to verify whether the predicted dose aligned with historical expert prescriptions. Furthermore, we conducted clinical simulations in the independent test set to evaluate dose optimization for suboptimal groups. These targets represent the safety thresholds of the optimal therapeutic window. Finally, this predictive framework was deployed as an accessible web interface named the “Gn Calculator.” An overarching visual summary of the study design, the predictive modeling framework, and its clinical benefit in optimizing Gn dosing is presented in Fig. 1.

This flowchart illustrates the systematic approach from initial patient screening to the development of the target-driven random forest model and highlights its clinical utility in navigating suboptimal patients toward an optimal therapeutic window.

### Statistical analysis

Previous research has demonstrated that optimal fresh embryo transfer outcomes in the PCOS population are achieved with 7–16 oocytes [6]. Therefore, we identified 7–16 oocytes as the optimal number for PCOS patients and categorized the participants into three groups: oocytes < 7 (low oocyte group), 7 ≤ oocytes ≤ 16 (optimal oocyte group), and oocytes > 16 (high oocyte group). Differences in baseline characteristics, COS parameters, and reproductive outcomes among the groups were assessed using Kruskal-Wallis, ANOVA, and chi-square tests as appropriate. For multiple group comparisons, post-hoc analyses were performed using Tukey’s HSD test (following ANOVA), Dunn’s test with Bonferroni correction (following Kruskal-Wallis), and pairwise chi-square tests with Bonferroni correction (for categorical variables). Based on these three predefined categories, we further evaluated the time to live birth and healthcare costs using decision tree models (detailed in Section S3 of the SA). Subsequently, to investigate specific metabolic impacts, patient classifications based on World Health Organization (WHO) BMI criteria and the Spearman correlation analysis between BMI and predicted Gn starting doses were comprehensively evaluated (detailed in Section S4 of the SA). All statistical analyses were conducted using R software (version 4.5.3), with *P* < 0.05 considered statistically significant.

## Results

### Baseline characteristics of different oocyte groups for PCOS women

Baseline characteristics are summarized in Table 1. The cohort comprised 976 women with a mean age of 31.09 ± 3.60 years (median 31.00 years, interquartile range [IQR] 29.00–33.00) and a mean BMI of 25.36 ± 3.97 kg/m² (median 25.39 kg/m², IQR 22.45–27.89). The cohort was stratified into three groups based on total oocyte yield: low oocyte group (*n* = 183), optimal oocyte group (*n* = 498), and high oocyte group (*n* = 295). While the mean age remained comparable across the three groups, significant differences were observed in BMI and ovarian reserve markers: AMH, AFC. Post-hoc pairwise comparisons revealed that the low oocyte group exhibited a significantly higher BMI compared to the high oocyte group. Conversely, the high oocyte group demonstrated the highest AMH and AFC levels among all groups.

**Table 1:** Baseline characteristics and IVF-ET data of women in different oocyte groups

Variable	Oocytes			*P*-value
	Low oocyte group (oocytes < 7) (*N* = 183)	Optimal oocyte group (7 ≤ oocytes ≤ 16) (*N* = 498)	High oocyte group (oocytes > 16) (*N* = 295)	
Age (years)	31.10 ± 3.97	31.03 ± 3.48	31.15 ± 3.55	0.903
BMI (kg/m^2^)	26.07 ± 3.95^a^	25.40 ± 4.15	24.83 ± 3.59^a^	0.004*
Fasting plasma glucose (mmol/L)	5.06 (4.07–11.45)	4.96 (3.67–15.05)	5.01 (2.77–14.25)	0.055
Fasting insulin (µU/mL)	14.58 (3.53–96.78)	16.59 (3.53–143.70)	14.93 (3.51–113.10)	0.049*
Insulin resistance [%(n/N)]	66.12% (121/183)	70.48% (351/498)	65.08% (192/295)	0.239
Infertility years (years)	4.00 (0.50–17.00)	4.00 (0.10–17.50)	3.00 (0.10–15.00)	0.145
Type of infertility [%(n/N)]				0.858
Primary	60.66% (111/183)	60.44% (301/498)	62.37% (184/295)	
Secondary	39.34% (72/183)	39.56% (197/498)	37.63% (111/295)	
Menstrual abnormalities [%(n/N)]	75.40% (138/183)	76.70% (382/498)	74.23% (219/295)	0.731
Severe male factor [%(n/N)]	0.55% (1/183)	2.61% (13/498)	2.37% (7/295)	0.246
AFC (n)	29 (8–93)^b^	31 (8–85)^f^	37 (10–80)^b, f^	< 0.001*
AMH (ng/ml)	7.75 (0.63–19.72)^a^	7.90 (0.69–19.84)^f^	9.47 (0.30-18.91)^a, f^	< 0.001*
bFSH (mIU/ml)	6.35 (1.24–9.99)^b^	6.01 (0.15–9.78)^f^	5.61 (1.87–9.51)^b, f^	< 0.001*
LH (mIU/ml)	6.27 (0.36–24.33)^c^	7.06 (0.71–48.42)^c^	7.06 (0.97–32.53)	0.032*
E_2_ (pg/ml)	35.73 (6.71–139.90)	37.04 (4.00-196.00)	36.19 (4.00-190.67)	0.824
T (ng/ml)	0.38 (0.06–1.34)	0.39 (0.00-2.30)	0.41 (0.02–1.31)	0.662
P (ng/ml)	0.41 (0.02–1.44)	0.40 (0.00-1.41)	0.43 (0.02–1.42)	0.650
PRL (ng/ml)	13.60 (0.00-63.40)^a, c^	15.35 (0.00-362.90)^c^	15.65 (0.00-111.50)^a^	0.012*

The homeostatic model assessment for insulin resistance (HOMA-IR) was calculated as [Fasting plasma glucose (FPG, mmol/l) * fasting insulin (FINS, µU/ml)] / 22.5, with values greater than 2.69 indicating insulin resistance

*PCOS* Polycystic ovary syndrome, *AFC* Antral follicle count, *BMI* Body mass index, *bFSH* basal follicle-stimulating hormone, *LH* Luteinizing hormone, *T* testosterone, *E*_2_Estradiol, *P* Progesterone, *PRL* Prolactin, *AMH* Anti-Müllerian hormone

Continuous variables obeying the normal distribution are expressed as mean ± standard deviation and were analyzed using the ANOVA test, followed by Tukey’s HSD test for post-hoc comparisons. Continuous variables obeying the skewed distribution are shown as the median (range) and were analyzed using the Kruskal-Wallis test, followed by Dunn’s test for post-hoc comparisons. Categorical variables are shown as percentages (number) and were analyzed using the chi-square test, followed by pairwise chi-square tests with Bonferroni correction for post-hoc comparisons. *P<0.05 was considered statistically significant. Post-hoc pairwise comparisons: ^a^: *P*<0.05, ^b^: *P*<0.001 between the low and high oocyte groups. ^c^:*P*<0.05, ^d^: *P*<0.001 between the low and optimal oocyte groups.^e^: *P*<0.05, ^f^: *P*<0.001 between the optimal and high oocyte groups

### COS and IVF-ET outcomes of different oocyte groups

As presented in Table 2, COS parameters and embryological outcomes exhibited significant variations across the three groups. Notably, OS increased progressively across the low, optimal, and high oocyte-yield groups.

The clinical pregnancy and live birth rates of fresh embryo transfer were relatively high in the low and optimal oocyte groups but were substantially lower in the high oocyte group. Furthermore, compared to the optimal and high oocyte groups, the low oocyte group required the lowest Gn starting dose; nevertheless, despite having the longest stimulation period and consuming the highest total Gn dose, this group yielded the lowest number of oocytes, alongside the poorest CPR and CLBR. Even though the high oocyte group achieved the highest CPR and CLBR, 96.61% of their fresh embryo transfer cycles were cancelled, and they experienced the highest OHSS incidence among the three groups. Therefore, these differences profoundly impacted the overall treatment burden (Table 2, as detailed in Supplementary Table S1). The low oocyte group incurred the highest average cost per live birth among the three groups, while the high oocyte group experienced the longest average time to achieve a live birth. Ultimately, the optimal oocyte group demonstrated both temporal and economic advantages, with an average time to live birth 62 days shorter than that of the high oocyte group and an average cost of ¥18,030.51 lower than the low oocyte group.

**Table 2:** Characteristics of IVF/ICSI treatment and outcomes for women in different oocyte retrieval groups

Variable	Oocytes			*P*-value
	Low oocyte group (oocytes < 7) (*N* = 183)	Optimal oocyte group (7 ≤ oocytes ≤ 16) (*N* = 498)	High oocyte group (oocytes > 16) (*N* = 295)	
Gn starting dose (IU)	168.85 ± 48.95^c^	178.86 ± 48.49^c^	178.81 ± 42.04	0.034*
Duration of stimulation (days)	9.83 ± 1.97^b, d^	9.05 ± 2.00^d^	8.74 ± 1.40^b^	< 0.001*
Gn total dose (IU)	1823 ± 583.62^b^	1721.91 ± 705.97^e^	1576.92 ± 502.95^b, e^	< 0.001*
Oocytes (n)	4.19 ± 1.52^b, d^	11.42 ± 2.86^d, f^	22.72 ± 6.53^b, f^	< 0.001*
OS	0.03 ± 0.01^b, d^	0.07 ± 0.03^d, f^	0.16 ± 0.06^b, f^	< 0.001*
Number of MII oocyte	3.58 ± 1.61^b, d^	9.40 ± 3.08^d, f^	18.38 ± 6.17^b, f^	< 0.001*
Number of 2PN	3.00 ± 1.48^b, d^	7.93 ± 3.00^d, f^	14.73 ± 5.19^b, f^	< 0.001*
Number of D3 embryo	2 (0–7)^b, d^	7 (0–16)^d, f^	13 (0–32)^b, f^	< 0.001*
Number of good-quality D3 embryo	1 (0–5)^b, d^	3 (0–14)^d, f^	5 (0–19)^b, f^	< 0.001*
Rate of good-quality D3 embryo [%(n/N)]	48.56% (237/488)^a, c^	50.70% (1809/3568)^c^	48.53% (1720/3543)^a^	0.007*
Number of blastocyst	0 (0–5)^b, d^	3 (0–14)^d, f^	7 (0–28)^b, f^	< 0.001*
Number of good-quality blastocyst	0 (0–3)^b, d^	1 (0–3)^d, f^	2 (0–17)^b, f^	< 0.001*
Rate of good-quality blastocyst [%(n/N)]	30.21% (42/139)^b, d^	34.04% (639/1877)^d^	31.29% (721/2304)^b^	< 0.001*
Rate of fresh ET cancellation [%(n/N)]	35.51% (65/183)^b, d^	55.22% (275/498)^d, f^	96.61% (285/295)^b, f^	< 0.001*
Rate of clinical pregnancy after fresh embryo transfer [%(n/N)]	39.83% (47/118)	44.39% (99/223)	30% (3/10)	0.519
Rate of live birth after fresh embryo transfer [%(n/N)]	25.42% (30/118)	27.35% (61/223)	10% (1/10)	0.461
CPR [%(n/N)]	62.29% (114/183)^b, c^	75.70% (377/498)^c, e^	83.72% (247/295)^b, e^	< 0.001*
CLBR [%(n/N)]	39.89% (73/183)^b^	50.20% (250/498)^f^	64.06% (189/295)^b, f^	< 0.001*
OHSS [%(n/N)]	0.55% (1/183)^a^	3.20% (16/498)	6.70% (20/295)^a^	0.002*
Time to live birth (days)	396.31 ± 71.80	399.99 ± 72.56^f^	462.35 ± 195.39^f^	< 0.001*
Healthcare costs to live birth (¥)	93,931.07^b, d^	75,900.56^d, f^	69,295.42^b, f^	< 0.001*

*COS* Controlled ovarian stimulation, *OS* Ovarian sensitivity, *OHSS* Ovarian hyperstimulation syndrome, *2PN* 2 pronuclei, *IVF-ET* in vitro fertilization-embryo transfer, *CPR* Cumulative pregnancy rate, *CLBR* Cumulative live birth rate

Continuous variables obeying the normal distribution are expressed as mean ± standard deviation and were analyzed using the ANOVA test, followed by Tukey’s HSD test for post-hoc comparisons. Continuous variables obeying the skewed distribution are shown as the median (range) and were analyzed using the Kruskal-Wallis test, followed by Dunn’s test for post-hoc comparisons. Categorical variables are shown as percentages (number) and were analyzed using the chi-square test, followed by pairwise chi-square tests with Bonferroni correction for post-hoc comparisons. *P < 0.05 was considered statistically significant. Post-hoc pairwise comparisons: ^a^: *P* < 0.05, ^b^: *P* < 0.001 between the low and high oocyte groups. ^c^: *P* < 0.05, ^d^: *P* < 0.001 between the low and optimal oocyte groups. ^e^: *P* < 0.05, ^f^: *P* < 0.001 between the optimal and high oocyte groups

### Feature selection and model construction driven by ovarian sensitivity

**Fig. 2:**
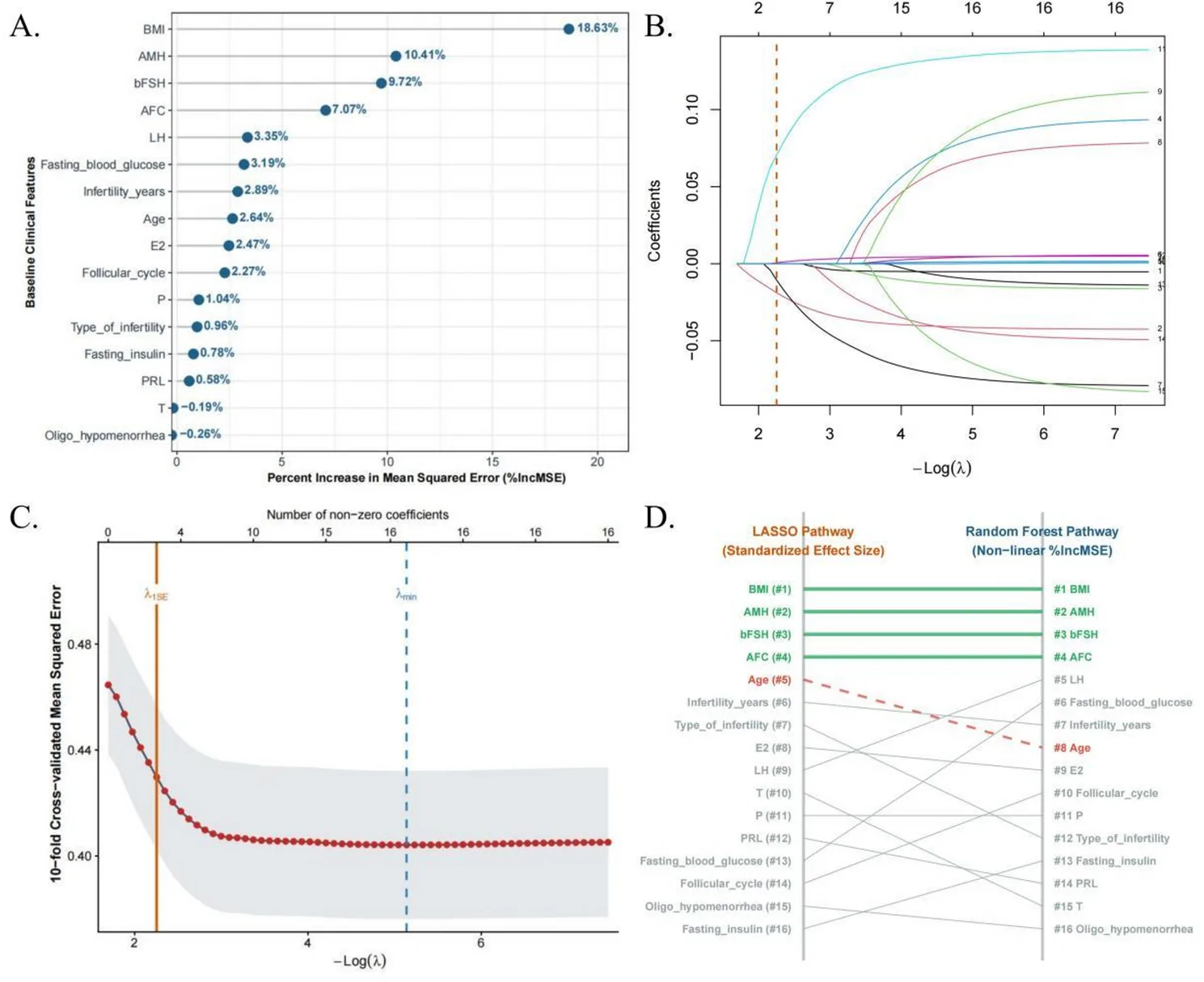
Feature selection driven by ovarian sensitivity (OS). **A** Random forest permutation importance for 16 candidate variables, expressed as the percentage increase in mean squared error (%IncMSE); larger values indicate greater predictive importance. **B** Least absolute shrinkage and selection operator (LASSO) coefficient paths for the 16 standardized variables. Each colored curve represents one variable; its position relative to zero indicates the direction and strength of association with log-transformed OS. Moving right indicates weaker penalization, allowing more coefficients to deviate from zero. The upper numbers indicate the number of non-zero coefficients, and the orange dashed line marks the λ value selected by the 1-standard-error (1-SE) rule. **C** Ten-fold cross-validation for selecting λ. Red points show the mean cross-validated mean squared error, and the gray band shows ±1 standard error. The orange solid and blue dashed lines indicate λ1SE and λmin, respectively; λ1SE was selected to obtain a more parsimonious model. **D** Rank-alignment slopegraph comparing the RF and LASSO rankings. The two methods converged on four core predictors: BMI, AMH, bFSH, and AFC

To balance algorithmic stability with clinical applicability, two machine learning methods focusing on OS were employed for variable selection. Initially, an RF algorithm ranked the predictive importance of all 16 candidate baseline variables, revealing that BMI exerted the most substantial predictive impact (Fig. 2A, Supplementary Table S2). Concurrently, the LASSO regression was applied to the same 16 variables to identify a stable and parsimonious subset of non-zero predictors, which consistently identified BMI as the most heavily weighted feature (Fig. 2B and C, Supplementary Table S3). A rank-alignment slopegraph was constructed to visualize the convergence of these two distinct algorithms (Fig. 2D). The intersection of the top-ranked features from the non-linear RF model and the linear LASSO regression consensus yielded four core predictors: BMI (ranked first), AMH, bFSH, and AFC. Additionally, correlation analyses confirmed the absence of severe multicollinearity among the four predictors (maximum *r* = 0.33), verifying their independent contributions (eFigure 5).

To evaluate predictive performance and justify the selection of the streamlined model, a series of RF regression models were developed to predict OS. As shown in Table 3, model performance remained stable throughout the sequential dimensionality reduction from the full 16 variables to 8 variables, and finally to the 4 core consensus variables. In the independent test set, the full 16-variable model achieved an R^2^ of 0.883 (95% CI: 0.844, 0.915), an RMSE of 0.237 (95% CI: 0.204, 0.270), and an MAE of 0.126 (95% CI: 0.113, 0.141). Notably, the streamlined 4-variable model maintained robust predictive capacity, yielding an R^2^ of 0.860 (95% CI: 0.809, 0.901), an RMSE of 0.254 (95% CI: 0.218, 0.296), and an MAE of 0.141 (95% CI: 0.127, 0.156). Calibration analysis on the independent test set showed high consistency between predicted and actual OS, confirming the absence of systematic bias (eFigure 4). Consequently, this 4-variable core model was selected to estimate observed OS across the patient cohort.

**Table 3:** Performance comparison of random forest models for predicting OS using varying numbers of clinical features

Model Complexity	Data set	*R*^2^ (95% CI)	RMSE (95% CI)	MAE (95% CI)
16 Variables Model	Training set	0.992 (0.990, 0.993)	0.082 (0.077, 0.088)	0.050 (0.048, 0.053)
	Validation set	0.892 (0.858, 0.924)	0.249 (0.211, 0.284)	0.130 (0.116, 0.146)
	Test set	0.883 (0.844, 0.915)	0.237 (0.204, 0.270)	0.126 (0.113, 0.141)
8 Variables Model	Training set	0.990 (0.988, 0.991)	0.088 (0.082, 0.095)	0.054 (0.051, 0.057)
	Validation set	0.891 (0.859, 0.922)	0.250 (0.213, 0.283)	0.135 (0.120, 0.150)
	Test set	0.882 (0.846, 0.915)	0.236 (0.204, 0.269)	0.129 (0.116, 0.144)
4 Variables Model	Training set	0.987 (0.984, 0.988)	0.099 (0.093, 0.106)	0.062 (0.059, 0.065)
	Validation set	0.858 (0.804, 0.908)	0.278 (0.226, 0.328)	0.148 (0.132, 0.165)
	Test set	0.860 (0.809, 0.901)	0.254 (0.218, 0.296)	0.141 (0.127, 0.156)

*R*^2^Coefficient of determination, *OS* Ovarian sensitivity, *RMSE* Root mean square error, *MAE* mean absolute error, *CI* Confidence interval

Data are presented as estimate (95% CI). 95% confidence intervals were calculated using the bootstrap method with 1,000 resamples. The 16-variable model includes all baseline candidate features ranked by importance: BMI, AMH, bFSH, AFC, LH, fasting plasma glucose, infertility years, age, E_2_, follicular cycle, P, type of infertility, fasting insulin, PRL, T, and menstrual abnormalities. The 8-variable model includes the top eight features identified by the random forest algorithm: BMI, AMH, bFSH, AFC, LH, fasting plasma glucose, infertility years, and age. The 4-variable model includes the core consensus predictors: BMI, AMH, bFSH, and AFC

### Prediction and optimization of Gn starting doses

**Fig. 3:**
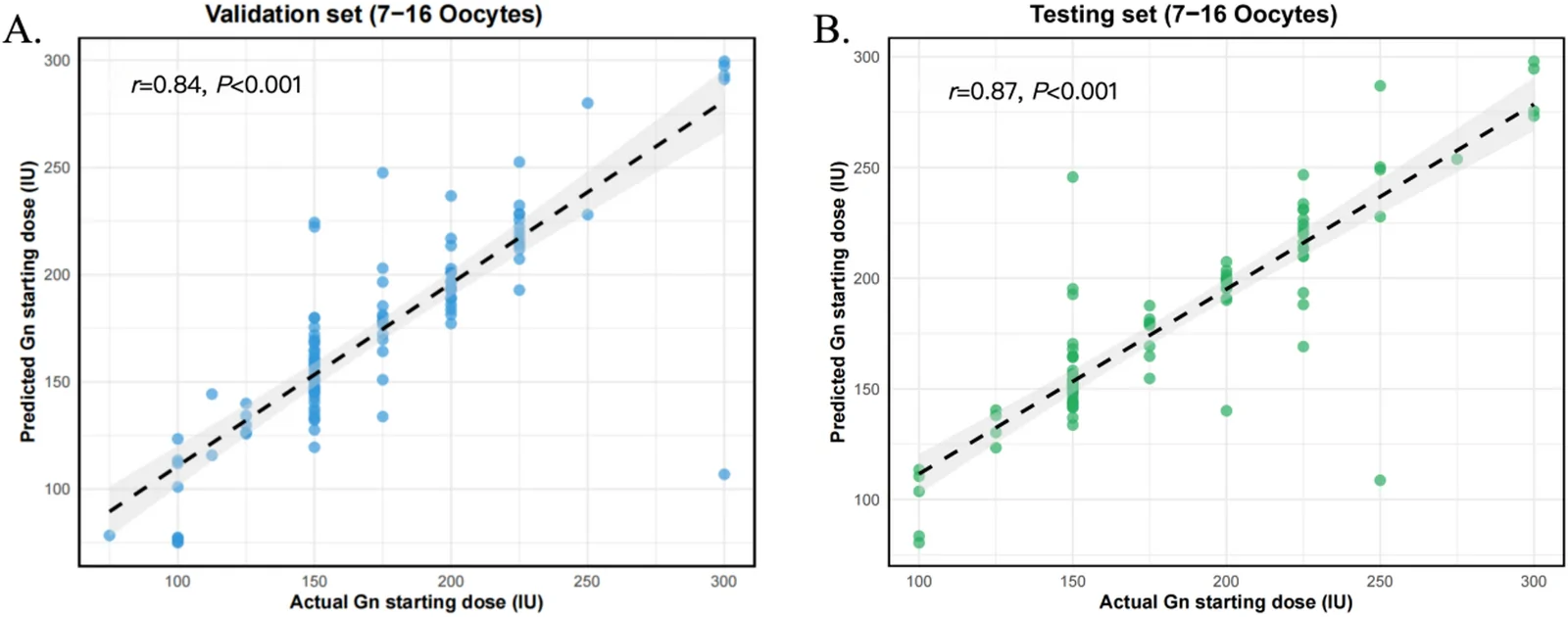
Predictive model accuracy for Gn starting doses in the optimal group. Actual oocyte yield served as the target to assess concordance between historical prescriptions and model predictions. **A** Correlation in the validation set (*r* = 0.84, *P* < 0.001). **B** Correlation in the independent test set (*r* = 0.87, *P* < 0.001)

The model functions as a dynamic navigator, translating observed OS into individualized Gn starting doses. The core premise of this approach is that when historical decisions yielded optimal clinical outcomes, a robust predictive model should highly replicate the historical prescriptions of expert physicians. Therefore, we specifically evaluated dose concordance within the optimal group (7–16 oocytes). Model-predicted doses exhibited strong linear correlations with historical expert prescriptions, with Pearson correlation coefficients (*r*) of 0.84 in the validation set and 0.87 in the independent test set (*P* < 0.001; Fig. 3).

**Fig. 4:**
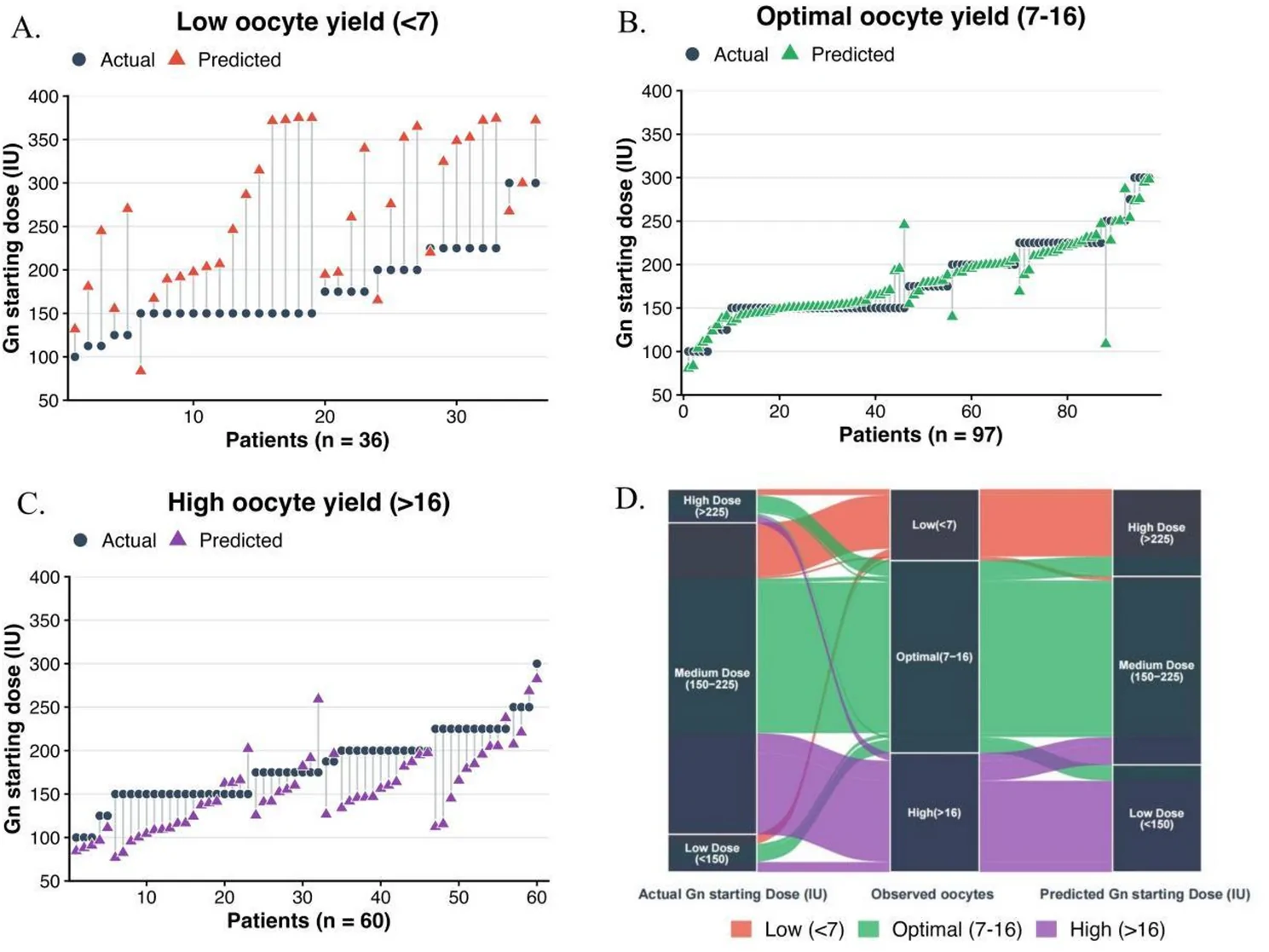
Model-guided Gn starting dose optimization in the independent test set. The target oocyte count was set to 7 for low-yield cycles (<7 oocytes) and 16 for high-yield cycles (> 16 oocytes). For cycles in the optimal range (7-16 oocytes), the observed oocyte yield was used as the target solely to assess dose concordance. In panels A-C, dark circles represent the actual Gn starting dose, colored triangles represent the predicted dose, and each gray line connects the two values for the same patient. **A** Low-yield group (< 7 oocytes), in which predicted doses were predominantly higher than actual doses. **B** Optimal group (7-16 oocytes), showing concordance between actual and predicted doses rather than a proposed dose correction. **C** High-yield group (>16 oocytes), in which predicted doses were predominantly lower than actual doses. **D** Sankey diagram showing patient flows in the independent test set from the actual Gn dose category (left: <150, 150–225, or >225 IU), through the observed oocyte-yield group (middle: < 7, 7-16, or > 16 oocytes), to the predicted Gn dose category (right). Ribbon colors denote oocyte-yield groups, and ribbon widths represent the number of patients. All predicted doses shown are retrospective simulations and were not prospectively administered

To evaluate clinical applicability, we simulated dose optimization in the independent test set by targeting the safety thresholds of the optimal therapeutic window (a target yield of 7 oocytes for the low group and 16 oocytes for the high group), while the actual oocyte yield was utilized as the customized target for the optimal group to evaluate the baseline concordance of the model in ideal clinical settings. Consequently, for this optimal group, the model-recommended doses closely matched historical prescriptions, confirming dose stability with a minimal MAE of 12.45 ± 19.68 IU (Fig. 4B). In contrast, the model systematically intervened to rectify dosing biases in suboptimal groups. For patients with low oocyte yield (< 7), the algorithm yielded a mean absolute dose deviation of 97.17 ± 67.79 IU (Fig. 4A). For patients with high oocyte yield (> 16), the model generated a mean absolute deviation of 35.71 ± 24.75 IU to effectively mitigate hyper-response risks (Fig. 4C).

These corrective interventions defined a clear dose-redistribution pattern, visualized via a three-axis Sankey diagram (Fig. 4D). This visualization maps the transformation of historical dosing patterns into model-optimized therapeutic strategies. Data from the Sankey diagram indicate a systematic relocation of patients from inappropriate dose tiers into optimal windows, demonstrating the capacity of the model to align starting doses with specific oocyte yield targets.

To evaluate physiological plausibility, we also assessed the relationship between BMI and Gn requirements. The model systematically escalated Gn doses across increasing BMI categories, confirming that the predictions were consistent with clinical physiology (Supplementary Section S4 and eFigure 6 in the SA).

### Online tool: “Gn Calculator”

**Fig. 5:**
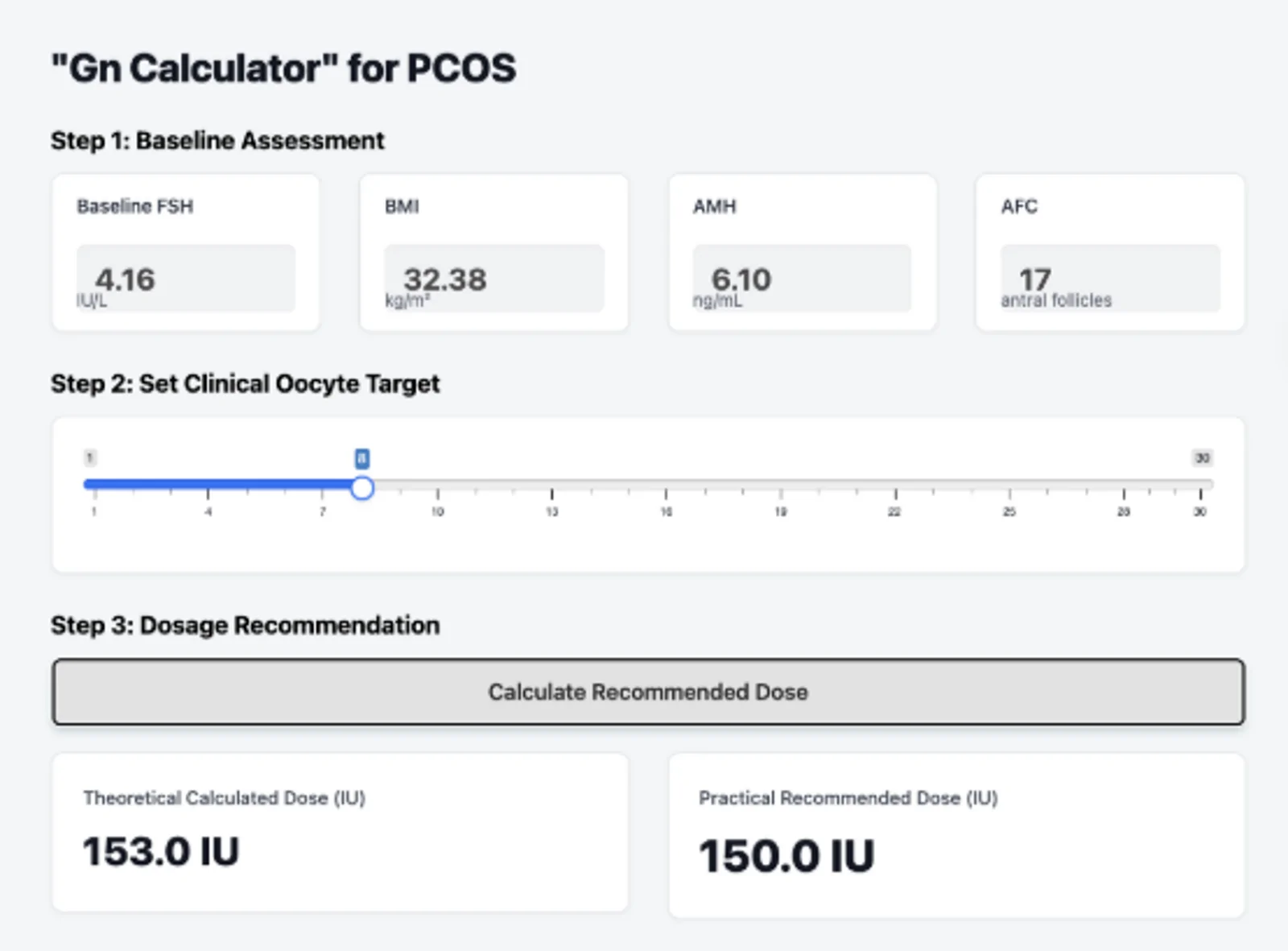
Online tool: Gn Calculator. The tool allows input of four baseline parameters (BMI, AMH, bFSH, and AFC) and a target oocyte yield. The backend RF algorithm computes predicted ovarian sensitivity and outputs a goal-directed Gn starting dose

The RF prediction model was deployed as an accessible web interface, the Gn Calculator (http://101.201.30.141:3838/app3/, Fig. 5). To determine individualized Gn starting doses, clinicians input four baseline metrics: BMI, AMH, bFSH, and AFC, along with a target oocyte yield. The algorithm computes the result instantaneously, providing goal-directed dosing recommendations to support clinical decision-making.

## Discussion

This study developed a target-driven machine learning model to estimate individualized Gn starting dose for women with PCOS undergoing COS. Using four routinely available variables (BMI, AMH, bFSH, and AFC), the model predicted observed ovarian sensitivity as a continuous response metric and then derived the corresponding Gn starting dose according to a predefined oocyte-yield target. The main value of this framework is not to prove that model-guided dosing has already improved clinical outcomes, but to provide a clinically interpretable and internally validated approach for individualized starting-dose estimation in PCOS patients. The model has been integrated into an online “Gn Calculator”, which may serve as a practical decision-support prototype for clinicians when selecting starting dose.

Our data support the finding that the 7–16 oocyte range represents a clinically balanced target-yield category, balancing the dual concerns of insufficient follicular recruitment and OHSS-related complications. A key methodological innovation of this study is how it navigates patients toward this optimal window: rather than directly predicting historical physician-prescribed Gn doses, the model estimates OS. In this study, OS was treated as an observed ovarian-response metric, providing a bridge between baseline clinical characteristics and Gn requirements. Compared with directly modeling historical prescriptions, this target-driven pathway may reduce the risk of simply reproducing empirical prescribing patterns and may better align stimulation intensity to ensure it is individualized according to expected ovarian responsiveness.

A further strength of this study is the balance between predictive performance and clinical parsimony. While clinical responses to Gn are rarely strictly linear, which is a limitation of previous nomograms [18, 19, 24, 26], excessively complex non-linear algorithms increase the data collection burden and limit bedside feasibility [27]. The streamlined four-variable RF model was designed to address these barriers, achieving an R^2^ of 0.860 (95% CI: 0.809, 0.901) in the independent test set while remaining highly accessible.

The selected predictors are clinically plausible and capture the profound heterogeneity of PCOS. BMI ranked highly in the model, exhibiting a positive correlation with predicted Gn requirements (*r* = 0.35, *P* < 0.001; eFigure 6B). This is consistent with pharmacokinetic principles, because higher BMI may increase Gn dose requirements. Because obesity alters ovarian responsiveness and is associated with prolonged stimulation and poorer embryo quality [28–30], integrating BMI alongside traditional ovarian reserve markers (AMH, AFC, and bFSH) may help capture the influence of metabolic status on Gn requirements. AMH and AFC are established markers of ovarian reserve and follicular recruitment potential [31]. The normal BMI group demonstrated higher AMH levels compared to the obesity group, a finding consistent with previous studies showing a negative correlation between AMH and BMI among women with PCOS [32, 33]. Furthermore, high AMH levels directly reflect ovarian reserve, while low AFC levels are associated with reduced ovarian response and poor pregnancy outcomes [34, 35]. Although predicting ovarian reserve by AFC is standard practice, ultrasound measurement accuracy is limited by potential human errors. Clinical practice reveals inconsistencies between AMH and AFC parameters in up to 20% of women, which supports the necessity of using both markers simultaneously in the current model [36]. Importantly, bFSH levels correlate inversely with ovarian reserve and indicate stimulation response [37]. The integration of these four variables ensures a comprehensive and physiological assessment for individualized dosing.

Conceptually, the target-oriented pathway in this study is consistent with the oocyte-number-oriented framework emphasized by the POSEIDON concept [11], although the present model was developed for women with PCOS rather than for low-prognosis patients. The potential utility of this approach was explored in simulated dose-adjustment analyses: the algorithm proactively generated step-up recommendations for patients with below-target oocyte yields and step-down recommendations for patients with above-target oocyte yields. These findings suggest that the model-derived recommendations are directionally consistent with clinical expectations. However, these analyses should be interpreted as post-hoc and simulation evidence rather than proof of clinical efficacy. Whether model-guided dosing can increase the probability of achieving the target oocyte range, reduce OHSS risk, preserve fresh embryo transfer opportunities, or reduce treatment costs requires prospective validation.

This study has several limitations. First, female age was not retained as a final predictor. Although age is a critical determinant of ovarian reserve and reproductive outcomes, nearly 90% of patients in this PCOS cohort were between 26 and 35 years of age, a range in which ovarian function may show relatively limited variation (Table S6). Therefore, the absence of age in the final model should not be interpreted as evidence that age is clinically unimportant. Second, this was a retrospective cohort study, and residual confounding, selection bias, and center-specific practice patterns cannot be fully excluded. Third, OS was an observed ovarian-response metric derived from oocyte yield and Gn starting dose, rather than a pure intrinsic biological marker. While the RF model captured non-linear relationships, deriving doses via the ratio formula assumes a proportional relationship. This practical framework performs reliably within the therapeutic window but simplifies complex receptor-saturation dynamics in extreme responders. Fourth, the 7–16 oocyte target range may not be generalizable to all PCOS phenotypes, age groups, BMI strata, stimulation protocols, or clinical settings. Fifth, the discussed clinical implications, including reduced OHSS risk, improved reproductive efficiency, shorter time to live birth, or lower costs, remain hypotheses generated from retrospective associations and simulation analyses rather than established clinical benefits from prospective trials. Finally, the current model estimates only the initial Gn starting dose and does not incorporate dynamic dose adjustments during stimulation. Therefore, multicenter prospective studies, ideally including external validation and randomized evaluation of model-assisted dosing, are needed to confirm the practical efficacy and safety of this framework. Future research should also explore whether dynamic Gn adjustment strategies can further improve individualized COS management.

## Conclusions

In conclusion, this study developed and internally validated an RF model to estimate individualized Gn starting doses for women with PCOS undergoing COS. By integrating observed OS, a predefined oocyte-yield target, and four routinely available clinical predictors, this framework provides a practical approach to individualized dose estimation. The online “Gn Calculator” may facilitate clinical decision support after prospective validation. Further multicenter and prospective studies are required to evaluate whether model-guided dosing can improve ovarian response, safety, and reproductive outcomes in real-world clinical practice.

## Supplementary Information

Supplementary Material 1.

## Data Availability

The datasets employed in this study are accessible from the corresponding author upon reasonable request (Qun Lu, E-mail: luqun1023@sina.com).

## Abbreviations

2PN: 2 Pronuclei
AFC: Antral Follicle Count
AMH: Anti-Müllerian Hormone
bFSH: Basal Follicle-Stimulating Hormone
BMI: Body Mass Index
CI: Confidence Interval
CLBR: Cumulative Live Birth Rate
COS: Controlled Ovarian Stimulation
CPR: Cumulative Pregnancy Rate
E2: Estradiol
FET: Frozen-Thawed Embryo Transfer
FPG: Fasting Plasma Glucose
Gn: Gonadotropin
GnRH: Gonadotropin-Releasing Hormone
hCG: Human Chorionic Gonadotropin
HMG: Human Menopausal Gonadotropin
HOMA-IR: Homeostatic Model Assessment for Insulin Resistance
ICSI: Intracytoplasmic Sperm Injection
IQR: Interquartile Range
IUI: Intrauterine Insemination
IVF-ET: In Vitro Fertilization and Embryo Transfer
LASSO: Least Absolute Shrinkage and Selection Operator
LH: Luteinizing Hormone
MAE: Mean Absolute Error
MSE: Mean Squared Error
OHSS: Ovarian Hyperstimulation Syndrome
OS: Ovarian Sensitivity
P: Progesterone
PCOS: Polycystic Ovary Syndrome
PRL: Prolactin
RF: Random Forest
RMSE: Root Mean Square Error
SA: Supplementary Appendix
T: Testosterone
