# Supplementary material for: Estimating individualized gonadotropin starting doses in women with PCOS: a target-driven machine learning model for GnRH-antagonist stimulation
Source: BMC Pregnancy Childbirth. 2026 Aug 10;26:1280. doi: 10.1186/s12884-026-09783-x (PMC13737509; doi:10.1186/s12884-026-09783-x)
Supplement: Supplementary file 1 [file 12884_2026_9783_MOESM1_ESM.docx]

Supplementary Appendix

Estimating Individualized Gonadotropin Starting Doses in Women with PCOS: A Target-Driven Machine Learning Model for GnRH-Antagonist Stimulation

Wenjia Meng ^a,1^, Ye Pan ^a,1^, Moxuan Zhang ^b,1^, Ziyi Song ^a,c^, Zerui Xiao ^c^, Rong Liang ^c^, Xiao Shi ^a^, Hongchu Bao ^d^, Lili Zhuang ^d*^, Qun Lu ^a*^

^a^ Medical Center for Human Reproduction, Beijing Chao-Yang Hospital, Capital Medical University, Beijing 100020, China

^b^ Beijing Neurosurgical Institute, Beijing Tiantan Hospital, Capital Medical University, Beijing, 100070, China

^c^ Reproductive Medical Center, Peking University People’s Hospital, Beijing 100044, P. R. China

^d^ Reproductive Medicine Centre, Yuhuangding Hospital of Yantai, Affiliated Hospital of Qingdao University, Yantai 264000, P. R. China

^1^ These three authors have equal contributions to this work

* Corresponding author:

Qun Lu, E-mail: [luqun1023@sina.com](mailto:luqun1023@sina.com) (Q. Lu)

Lili Zhuang, E-mail: lilizhuang06@126.com

**Material list:**

Section S1: The collection of patient baseline data

Section S2: Objective criteria to assess OHSS risk

Section S3: Assessment of healthcare costs

Section S4: Relationship between BMI and predicted Gn starting dose

Table S1 Cost estimation of IVF-ET.

Table S2 Ranking and percentage of characteristic importance for 16 candidate variables in random forest.

Table S3 Standardized Effect Size of the importance of the characteristics of the 16 candidate variables in LASSO regression.

Table S4 Grouping PCOS patients by BMI and comparing IVF-related parameters.

Table S5 Patients with changes between actual and predicted Gn starting dose by oocyte group.

Table S6 Age distribution of PCOS women.

Table S7 Trigger methods in different oocyte retrieval groups.

Table S8 The number and types of embryos transferred in fresh ET across different oocyte retrieval groups.
Table S9 Clinical characteristics information of PCOS women.

eFigure 1 Decision Tree Model for Low Oocyte Group.

eFigure 2 Decision Tree Model for Optimal Oocyte Group.

eFigure 3 Decision Tree Model for High Oocyte Group.

eFigure 4 Calibration curve of the random forest model on the independent test set.

eFigure 5 Correlation and joint distribution matrix of core predictors and ovarian sensitivity.

eFigure 6 Relationship between BMI and predicted Gn starting dose.

eFigure 7 Distribution of Reasons for Fresh ET Cancellation in Different Oocyte Retrieval Groups.

**Section S1: The collection of patient baseline data**

Baseline characteristics collected included age, BMI, type and duration of infertility, number of IVF-ET cycles, and menstrual abnormalities. Laboratory tests, including bFSH, luteinizing hormone (LH), E2, progesterone (P), testosterone (T), prolactin (PRL), and AMH levels, were conducted on days 2-4 of the menstrual cycle, as well as Fasting blood-glucose and fasting insulin. Menstrual abnormalities were defined as menstrual cycles <21 days or >35 days, drug-dependent menstruation or amenorrhea, and irregular uterine bleeding. Testosterone levels greater than 0.481ng/ml was regarded as hyperandrogenemia. AFC was obtained on days 2-4 of the menstrual cycle with transvaginal ultrasound measurements. Polycystic ovarian morphology (PCOM) was defined by the presence of 12 or more follicles in each ovary measuring 2-9 mm in diameter, and/or an increased ovarian volume (10 ml or more). The homeostatic model assessment for insulin resistance (HOMA-IR) was calculated as [Fasting blood-glucose (FPG,mmol/l)* fasting insulin (FINS, µU/ ml)] / 22.5, with values greater than 2.69 indicating insulin resistance.

**Section S2: Objective criteria to assess OHSS risk**

We followed the American Society for Reproductive Medicine (ASRM) guidelines for the prevention and diagnosis of moderate and severe OHSS in 2016 (1), which included PCOS, a history of OHSS, AFC > 24, and AMH level > 3.4 ng/mL; during the current COS cycle, risk factors included >17 follicles over 10 mm at the trigger, elevated estradiol levels at the trigger (>3,500 pg/mL), and >15 oocytes retrieved.

**References:**

1. Prevention and treatment of moderate and severe ovarian hyperstimulation syndrome: a guideline. Fertil Steril. 2016;106(7):1634-47.

**Section S3: Assessment of healthcare costs**

### Time to live birth and healthcare costs

We evaluated the average time from the commencement of infertility treatment to achieve a live birth across three groups. The calculation was as follows: approximately one month was required from the initial infertility examination to the start of the IVF cycle, about 15 days for the COS process, and we recorded the time from oocyte retrieval to the first live birth for each patient.

We developed a decision tree model using R software to calculate the average cost for each patient, including expenses from the initial infertility treatment examination, IVF cycles, and medical costs from pregnancy to delivery. Participants were categorized into three groups as follows: oocytes≤6 (Low oocyte group), 7≤oocytes≤16 (Optimal oocyte group), and oocytes≥17 (High oocyte group). The cost of IVF was separately estimated based on local expenses (Table S1 in SA). The simplified formula for calculating the average cost of obtaining a live birth across three groups was as follows:

$$Average cost=\frac{\sum Cost per patient}{Number of live births}$$

### Decision tree

A decision tree model was developed using R software (version 4.2.2) to calculate the average cost required to obtain a live birth for each patient in three groups. A decision tree is a decision model that simulates the probabilities, costs, and outcomes associated with treating a group of patients according to a predetermined method. The model simulates two transfer strategies: receiving fresh ET and whole embryo freezing. For each patient, there were three possible outcomes for each transfer: no further transfers without live birth, continued FET without live birth, and live birth. The decision tree model for the three groups is shown in eFigure 1-3.

### The IVF cost

The research focus of this study was the healthcare system, excluding non-medical costs, time costs, and productivity losses. This analysis reflects the direct medical costs of IVF treatment for infertility in China. We separately estimated the cost of IVF based on local expenses. Direct medical costs primarily included the expenses for examinations before patients received subfertility treatments and those incurred throughout the IVF cycle. According to the medical cost analysis of the hospital information system, direct medical costs for IVF were categorized into the costs of tests, medications, IVF procedures, and physician services. Medication costs encompassed the expenses for COS, endometrial preparation, and luteal phase support. Cost estimates for IVF procedures included egg retrieval, IVF laboratory procedures, hospitalization for OHSS, cryopreserved embryos, thawing procedures, and ET procedures. We also gathered direct medical costs associated with live births to estimate total costs. The expenses for prenatal visits and cesarean sections were considered part of the live birth costs. In this study, only direct medical costs were used for analysis due to the high variability and complexity of calculating indirect costs. All included costs (in CNY) are presented in Table S1.

### Definitions

*T_fresh_*: Total cost of fresh ET pathway

*T_frozen_*: Total cost of whole embryo freezing pathway

*C_fresh_*: Cost of fresh ET

*C_frozen_*: Cost of whole embryo freezing

*C_FET_*: Cost of FET

*C_delivery_*: Cost from pregnancy to delivery

*N_fresh_*: Number of fresh ET

*N_frozen_*: Number of whole embryo freezing

*N_i_FET fresh_*: Round *i* FET after fresh ET

*N_i_FET frozen_*: Round *i* FET after whole embryo freezing

*L*: Number of live births for all patients

*L_fresh_*: Total number of live births achieved by patients who chose the fresh ET pathway

*L_frozen_*: Total number of live births achieved by patients who chose the whole embryo freezing pathway

### Formulas

Fresh ET pathway:

$$T_{fresh}=N_{fresh}*C_{fresh} + \sum_{i=1}^{4} {i*N}_{i_{FET fresh}}* C_{FET} +L_{fresh} * C_{delivery}$$

Whole embryo freezing pathway:

$$T_{frozen}=N_{frozen}*C_{frozen}+ \sum_{i=1}^{4} i*N_{i_{FET frozen}}* C_{FET} +L_{frozen} * C_{delivery}$$

Total number of live births and Total cost:

$$L = L_{fresh} +L_{frozen}$$

$$Total cost=T_{fresh} +T_{frozen}$$

Average cost:

$$Average cost=\frac{Total cost}{L}$$

Based on the above definitions and formulas, we derived the average costs required to obtain a live birth for each of the three groups.

**Section S4:** Relationship between BMI and predicted Gn starting dose

To evaluate the clinical interpretability of the model, we analyzed the relationship between body mass index (BMI) and Gn requirements (eFigure 6). A positive correlation was observed between BMI and the actual Gn starting doses administered empirically by physicians (R = 0.34, *P* < 0.001; eFigure 6A). In contrast, the predicted Gn doses generated by the algorithm demonstrated a smooth, continuous positive correlation with BMI (R = 0.35, *P* < 0.001; eFigure 6B). For these predictions, the target oocyte count was set at 7 for patients with low yield (<7), 16 for patients with high yield (>16), and the actual retrieved oocyte count was utilized as the target for the optimal group (7 to 16). When patients were stratified by standard BMI categories, the model systematically escalated the predicted doses from the underweight group to the obesity group (eFigure 6C, *P* < 0.001). This dose-escalation pattern confirms that the algorithm effectively captured fundamental physiological rules rather than memorizing statistical noise.

Furthermore, a radar plot (eFigure 6D, detailed outcome comparisons are provided in Supplementary Table S4) illustrates the multi-dimensional clinical profiles across BMI categories. While the normal BMI cohort achieved optimal embryo outcomes with the lowest risk of ovarian hyperstimulation syndrome (OHSS), the obesity cohort required more intense Gn stimulation but yielded fewer optimal outcomes.

**Table S1 Cost estimation of IVF-ET.**

| Stage | Program | | Average Cost |
| --- | --- | --- | --- |
| pre-IVF | Cost of Examination | IVF laboratory procedure for female | ￥2,500 |
|  |  | IVF laboratory procedure for male | ￥2,000 |
|  | Cost of physician services |  | ￥500 |
| IVF | Cost of Drugs | COS ^a^ | Low oocyte group: ¥6,500  Optimal oocyte group: ¥6,000  High oocyte group: ¥5,500 |
|  | Cost of Examination | Laboratory testing | ￥1,000 |
|  |  | Ultrasonography | ￥500 |
|  | Cost of IVF procedure | Ovum pickup | ￥2,400 |
|  |  | In vitro fertilization and embryo culture | ￥4,698 |
|  |  | ICSI ^a^ | Low oocyte group: ¥804  Optimal oocyte group: ¥530  High oocyte group: ¥646 |
|  |  | Morphology evaluation of embryos | ￥410 |
|  |  | Costs of severe OHSS hospitalization ^b^ | ￥15000 |
|  |  | Cost of embryo freezing ^a^ | Low oocyte group: ¥3,431  Optimal oocyte group: ¥4,425  High oocyte group: ¥5,587 |
|  | Cost of physician services |  | ￥500 |
| fresh ET | Cost of Drugs | Luteal-phase support | ￥2,000 |
|  | Cost of Examination | Laboratory testing | ￥400 |
|  |  | Ultrasonography | ￥100 |
|  | Cost of IVF procedure | ET procedures | ￥2,300 |
|  | Cost of physician services |  | ￥100 |
| FET | Cost of Drugs | Endometrial preparation | ￥300 |
|  |  | Luteal-phase support | ￥2,000 |
|  | Cost of Examination | Laboratory testing | ￥400 |
|  |  | Ultrasonography | ￥100 |
|  | Cost of IVF procedure | Thawing procedure | ￥1,244 |
|  |  | ET procedures | ￥2,300 |
|  | Cost of physician services |  | ￥500 |
| Pregnancy | Cost of Examination | Prenatal examination | ￥4,000 |
|  | Cost of the delivery procedure | Vaginal delivery or Caesarean section | ￥6,333 |

^a^ Average costs for different groups.

^b^ The hospitalization cost for patients with severe OHSS was approximately ¥15,000 per person. The number of patients with severe OHSS in the low, optimal, and high oocyte groups was 0, 1, and 11, respectively.

**Table S2 Ranking and percentage of characteristic importance for 16 candidate variables in random forest.**

| **Ranking** | **Variable** | **Importance%** |
| --- | --- | --- |
| **1** | BMI (kg/m²) | 18.63% |
| **2** | AMH (ng/ml) | 10.41% |
| **3** | bFSH (mIU/ml) | 9.72% |
| **4** | AFC (n) | 7.07% |
| **5** | LH (mIU/ml) | 3.35% |
| **6** | Fasting plasma glucose (mmol/L) | 3.19% |
| **7** | Infertility years (years) | 2.89% |
| **8** | Age (years) | 2.64% |
| **9** | E2 (pg/ml) | 2.47% |
| **10** | Follicular cycle | 2.27% |
| **11** | P (ng/ml) | 1.04% |
| **12** | Type of infertility | 0.96% |
| **13** | Fasting insulin (μU/mL) | 0.78% |
| **14** | PRL (ng/ml) | 0.58% |
| **15** | T (ng/ml) | -0.19% |
| **16** | Menstrual abnormalities | -0.26% |

**Table S3 Standardized Effect Size of the importance of the characteristics of the 16 candidate variables in LASSO regression.**

| **Ranking** | **Variable** | **Standardized Effect Size** |
| --- | --- | --- |
| **1** | BMI (kg/m²) | 0.110210444167143 |
| **2** | AMH (ng/ml) | 0.069324963320532 |
| **3** | bFSH (mIU/ml) | 0.0217765966793253 |
| **4** | AFC (n) | 0.0134157978531984 |
| **5** | Age (years) | 0 |
| **6** | Infertility years (years) | 0 |
| **7** | Type of infertility | 0 |
| **8** | E2 (pg/ml) | 0 |
| **9** | LH (mIU/ml) | 0 |
| **10** | T (ng/ml) | 0 |
| **11** | P (ng/ml) | 0 |
| **12** | PRL (ng/ml) | 0 |
| **13** | Fasting plasma glucose (mmol/L) | 0 |
| **14** | Follicular cycle | 0 |
| **15** | Menstrual abnormalities | 0 |
| **16** | Fasting insulin (μU/mL) | 0 |

**Table S4 Grouping PCOS patients by BMI and comparing IVF-related parameters.**

| **Variable** | **BMI** | | | | *P***-value** |
| --- | --- | --- | --- | --- | --- |
|  | **Underweight (N=27)**  **<18.5 kg/m2** | **Normal (N=425)**  **18.5~24.9 kg/m2** | **Overweight (N=404) 25.0~29.9 kg/m2** | **Obesity (N=120)**  **≥30.0 kg/m2** |  |
| **bFSH (mIU/ml)** | 7.13 (1.87-9.78)^c,e^ | 5.96 (1.92-9.99) | 5.88 (1.24-9.87)^c^ | 5.87 (0.15-9.35)^e^ | 0.016* |
| **AMH (ng/ml)** | 8.00 (2.79-19.85) | 9.19 (0.30-19.54)^i^ | 8.34 (0.67-19.73) | 6.66 (0.70-19.48)^i^ | 0.003* |
| **AFC (n)** | 29.00 (12.00-44.00) | 32.00 (8.00-84.00) | 33.00 (8.00-85.00) | 31.50 (8.00-93.00) | 0.035* |
| **Gn starting dose (IU)** | 150 (75-225)^d,f^ | 150 (75-375)^h,j^ | 175 (75-300)^d,k^ | 200 (75-300)^f,j,k^ | <0.001* |
| **Predicted Gn starting dose**  **(IU)** | 148.4 (84.9-355.9)^d,f^ | 151.6 (75.0-375.0)^h,j^ | 181.8 (75.9-375.0)^d,l^ | 215.4 (89.3-374.9)^f,j,l^ | <0.001* |
| **Duration of stimulation (days)** | 9.00 (7-15) | 8.00 (3-22)^g,j^ | 9.00 (2-23)^g^ | 9.00 (7-16)^j^ | <0.001* |
| **Gn total dose (IU)** | 1400 (675-2600)^c,f^ | 1387.5 (300-5225)^h,j^ | 1700 (300-6450)^c,l^ | 1975 (1125-4700)^f,j,l^ | <0.001* |
| **Oocytes (n)** | 11.00 (3-34) | 13.00 (1-46)^j^ | 12.00 (1-50) | 11.00 (1-67)^j^ | 0.002* |
| **OS** | 0.08 (0.01-0.27)^e^ | 0.08 (0.01-0.41)^h,j^ | 0.07 (0.00-0.33)^e,l^ | 0.05 (0.01-0.45)^j,l^ | <0.001* |
| **Number of MII oocyte** | 9 (2-29) | 11 (0-39)^i^ | 10 (0-42) | 8 (0-61)^i^ | 0.002* |
| **Number of 2PN** | 7 (1-23) | 9 (0-30)^g,j^ | 8 (0-28)^g^ | 7 (0-48)^j^ | <0.001* |
| **Number of D3 embryo** | 6 (0-13) | 7 (0-32) | 7 (0-28) | 6 (0-23) | 0.055 |
| **Number of good-quality D3 embryo** | 3 (0-11) | 3 (0-19) | 3 (0-18) | 2 (0-14) | 0.333 |
| **Rate of good-quality D3 embryo [%(n/N)]** | 41.15% (93/226) | 40.33% (1683/4173) | 45.57% (1604/3520) | 41.42% (386/932) | 0.132 |
| **Number of blastocyst** | 3 (0-10) | 4 (0-21)^i^ | 3 (0-23) | 2 (0-28)^i^ | 0.005* |
| **Number of good-quality blastocyst** | 0 (0-6) | 1 (0-12) | 1 (0-17) | 0 (0-9) | 0.296 |
| **Rate of good-quality blastocyst [%(n/N)]** | 30.21% (29/96) | 30.36% (624/2055) | 35.59% (621/1745) | 30.19% (128/424) | 0.704 |
| **Rate of clinical pregnancy after fresh embryo transfer [%(n/N)]** | 80.00% (8/10) | 38.93% (51/131) | 42.04% (66/157) | 45.28% (24/53) | 0.086 |
| **Rate of live birth after fresh embryo transfer [%(n/N)]** | 50.00% (5/10) | 25.19% (33/131) | 25.48% (40/157) | 26.42% (14/53) | 0.387 |
| **CPR [%(n/N)]** | 77.78% (21/27) | 78.35% (333/425) | 75.25% (304/404) | 66.67% (80/120) | 0.185 |
| **CLBR [%(n/N)]** | 59.26% (16/27) | 57.41% (244/425)^i^ | 50.50% (204/404) | 40.00% (48/120)^i^ | 0.036* |
| **OHSS [%(n/N)]** | 3.70% (1/27) | 3.00% (13/425) | 4.70% (19/404) | 3.33% (4/120) | 0.640 |

BMI: body mass index; bFSH: basal follicle-stimulating hormone; AMH: anti-Müllerian hormone; AFC: antral follicle count; OS: ovarian sensitivity; OHSS: ovarian hyperstimulation syndrome; 2PN: 2 pronuclear; IVF-ET: in vitro fertilization-embryo transfer; CPR: cumulative pregnancy rate; CLBR: cumulative live birth rate;

Continuous variables obeying the normal distribution are expressed as mean±standard deviation and were analyzed using the ANOVA test, followed by Tukey’s HSD test for post-hoc comparisons. Continuous variables obeying the skewed distribution are shown as the median (range) and were analyzed using the Kruskal-Wallis test, followed by Dunn’s test for post-hoc comparisons. Categorical variables are shown as percentages (number) and were analyzed using the chi-square test, followed by pairwise chi-square tests with Bonferroni correction for post-hoc comparisons. *P<0.05 was considered statistically significant.

Post-hoc pairwise comparisons:

^a^: *P*<0.05, ^b^: *P*<0.001 between the underweight and normal groups.

^c^: *P*<0.05, ^d^: *P*<0.001 between the underweight and overweight groups.

^e^: *P*<0.05, ^f^: *P*<0.001 between the underweight and obesity groups.

^g^: *P*<0.05, ^h^: *P*<0.001 between the normal and overweight groups.

^i^: *P*<0.05, ^j^: *P*<0.001 between the normal and obesity groups.

^k^: *P*<0.05, ^l^: *P*<0.001 between the overweight and obesity groups.

**Table S5 Patients with changes between actual and predicted Gn starting dose by oocyte group.**

| **Dose difference (Actual minus Predicted)** | **Low oocyte group** | **Optimal oocyte group** | **High oocyte group** |
| --- | --- | --- | --- |
|  | **oocytes≤6** | **7≤oocytes≤16** | **oocytes≥17** |
|  | **(N=183)** | **(N=498)** | **(N=295)** |
| **Decrease** |  |  |  |
| patients [%(n/N)] | 69.39% (127/183) | 53.41% (266/498) | 49.49% (146/295) |
| Average Dose Change (IU) | -41.14±24.95 | -15.04±11.58 | -25.46±20.36 |
| **Increase** |  |  |  |
| patients [%(n/N)] | 30.60% (56/183) | 46.59% (232/498) | 50.51% (149/295) |
| Average Dose Change (IU) | 36.31±37.50 | 17.05±14.02 | 36.63±26.26 |

Average Dose Change: Negative values indicate that the actual dose is lower than the predicted dose, while positive values indicate that the actual dose is higher than the predicted dose.

**Table S6 Age distribution of PCOS women.**

| **Age Group** | **Percentage[%(n/N)]** |
| --- | --- |
| 20-25 | 3.89% (38/976) |
| 26-30 | 43.95% (429/976) |
| 31-35 | 42.01% (410/976) |
| 35-40 | 8.09% (79/976) |
| >40 | 2.05% (20/976) |

**Table S7 Trigger methods in different oocyte retrieval groups.**

| **Variable** | **Oocytes** | | | ***P*-value** |
| --- | --- | --- | --- | --- |
|  | **Low oocyte group** | **Optimal oocyte group** | **High oocyte group** |  |
|  | **oocytes≤6** | **7≤oocytes≤16** | **oocytes≥17** |  |
|  | **(N=183)** | **(N=498)** | **(N=295)** |  |
| **Triggering methods [%(n/N)]** |  |  |  | <0.001* |
| **hCG**  **6500-10000IU** | 71.58%  (131/183) ^b, d^ | 69.08%  (344/498) ^d, f^ | 34.58%  (102/295) ^b, f^ |  |
| **GnRHa 0.2mg + hCG 2000IU** | 25.14%  (46/183) ^b, d^ | 29.52%  (147/498) ^d, f^ | 62.71%  (185/295) ^b, f^ |  |
| **GnRHa 0.2mg** | 3.28%  (6/183) ^b, d^ | 1.40%  (7/498) ^d, f^ | 2.71%  (8/295) ^b, f^ |  |

**P*<0.05 was considered statistically different.

^b^ The low and high oocyte groups were considered statistically different (*P*<0.001).

^d^ The low and optimal oocyte groups were considered statistically different (*P*<0.001).

^f^ The optimal and high oocyte groups were considered statistically different (*P*<0.001).

**Table S8 The number and types of embryos transferred in fresh ET across different oocyte retrieval groups.**

| **Number** | **Types** | **Oocytes** | | | ***P*-value** |
| --- | --- | --- | --- | --- | --- |
|  |  | **Low oocyte group** | **Optimal oocyte group** | **High oocyte group** |  |
|  |  | **oocytes≤6** | **7≤oocytes≤16** | **oocytes≥17** |  |
|  |  | **(N=118)** | **(N=223)** | **(N=10)** |  |
| **One embryo** |  | 15.25% (18/118)^a^ | 10.76% (24/223)^f^ | 60.00% (6/10)^a,f^ | <0.001* |
|  | D3 embryo | 88.89% (16/18) | 87.50% (21/24) | 33.33%  (2/6) | NS |
|  | blastocyst | 11.11% (2/18) | 12.50% (3/24) | 66.67%  (4/6) | NS |
| **Two embryos** |  | 84.76% (100/118)^a^ | 89.24% (199/223)^f^ | 40.00% (4/10)^a,f^ | <0.001* |
|  | D3 embryo | 99.00% (99/100) | 98.99% (197/199) | 100.00% (4/4) | NS |
|  | blastocyst | 1.00% (1/100) | 1.01% (2/199) | 0.00% (0/4) | NS |

Continuous variables obeying the normal distribution are expressed as mean±standard deviation and were analyzed using the ANOVA test.

Continuous variables obeying the skewed distribution are shown as the median (range) and were analyzed using the Kruskal-Wallis test.

Categorical variables are shown as percentages (number) and were analyzed using the chi-square test.

**P*<0.05 was considered statistically different.

^a^ The Low and High oocyte groups were considered statistically different (*P*<0.05).

^f^ The Optimal and High oocyte groups were considered statistically different (*P*<0.001).

**Table S9 Clinical characteristics information of PCOS women.**

| **Clinical characteristics** | **Percentage[%(n/N)]** |
| --- | --- |
| Oligo-or anovulation | 97.23% (949/976) |
| Hyperandrogenism | 35.35% (345/976) |
| Polycystic ovarian morphology | 90.27% (881/976) |

**eFigure 1 Decision Tree Model for Low Oocyte Group.**

Success: refers to obtaining a live birth. Fail: refers to not obtaining a live birth.

FET: Frozen-Thawed Embryo Transfer.

**eFigure 2 Decision Tree Model for Optimal Oocyte Group.**

Success: refers to obtaining a live birth. Fail: refers to not obtaining a live birth.

FET: Frozen-Thawed Embryo Transfer.

**eFigure 3 Decision Tree Model for High Oocyte Group.**

Success: refers to obtaining a live birth. Fail: refers to not obtaining a live birth.

FET: Frozen-Thawed Embryo Transfer.

**eFigure 4. Calibration curve of the random forest model on the independent test set.**

The scatter plot demonstrates the agreement between actual and model-predicted log-transformed ovarian sensitivity ($\ln(OS)$). The red line with gray shading represents the linear fit and its 95% confidence interval, while the dashed line indicates the ideal reference (y=x). A slope of 1.114 and an intercept of 0.310 indicate robust algorithmic calibration without severe systemic over- or under-estimation.

**eFigure 5. Correlation and joint distribution matrix of core predictors and ovarian sensitivity.**

The upper right panels display Spearman correlation coefficients, with red and blue shading representing positive and negative correlations, respectively. The lower left panels utilize 2D density contour plots to visualize the joint distributions of variable pairs.

**eFigure 6 Relationship between BMI and predicted Gn starting dose.**

**A.B.** Correlation between BMI and the predicted Gn starting dose in the optimal oocyte group (A, *r* = 0.34, *P* < 0.001) and the overall patient population (B, *r* = 0.35, *P* < 0.001).

**C.** The model-recommended Gn starting dose escalates significantly with increasing BMI categories (*P* < 0.001).

**D.** A radar plot visualizing the multi-dimensional clinical phenotypes and IVF outcomes across different BMI cohorts. Parameters include the predicted Gn starting dose, total days of Gn stimulation, total Gn dose, number of retrieved oocytes, number of 2PN, and the incidence of OHSS. Values are normalized to a percentage scale (0%–100%) for comparative visualization. Note: To generate these predictions, the target oocyte count was set at 7 for patients with low yield (<7), 16 for patients with high yield (>16), and the actual retrieved oocyte count was utilized as the target for the optimal group (7 to 16). Detailed statistical comparisons are provided in Supplementary Table S4.

**eFigure 7 Distribution of Reasons for Fresh ET Cancellation in Different Oocyte Retrieval Groups.**
